# Breast cancer screening practices of African migrant women in Australia: a descriptive cross-sectional study

**DOI:** 10.1186/s12905-017-0384-0

**Published:** 2017-04-17

**Authors:** Olayide Oluyemisi Ogunsiji, Cannas Kwok, Lee Chun Fan

**Affiliations:** 1 Western Sydney University, Penrith, NSW Australia; 2 Singapore Clinical Research Institute School of Public Health, The University of Hong Kong, 5/F William MW Mong Block, 21 Sassoon Road, Hong Kong, China

**Keywords:** Breast cancer, Screening, African women, Australia, African migrant women, Migrant women, African-Australian

## Abstract

**Background:**

Breast cancer is the most diagnosed cancer among women and a leading cause of mortality and morbidity, globally. Breast cancer mortality can be improved through routine cancer screening, yet migrant populations have lower participation rates. While African migrants are among the fastest growing migrant population in Australia, their breast cancer screening behaviour is under-studied. The aims of this study were to report breast cancer screening status of African migrant women and factors associated with their breast cancer screening behaviour in Australia.

**Methods:**

A descriptive, cross-sectional approach was utilised for this study. Two hundred and sixty four African migrant women aged 18–69 years and recruited from a number of organisations responded to a self-reported African version of the Breast Cancer Screening Beliefs Questionnaire (BCSBQ). Main research variables are breast cancer screening practices and demographic characteristics and total scores on each of the BCSBQ subscales. Multivariable logistic regression analyses were performed to investigate the impact of the demographic variables on the likelihood of women in the target age range 50–74 years having screening practices as recommended.

**Results:**

While most of the participants heard of breast awareness (76.1%) and mammogram (85.2%), only 11.4% practised monthly breast awareness, whereas 65.9% had ever had a mammogram as frequently as recommended. Age and employment were determining factors for participating in mammogram. Significant different scores were found in the “Practical barriers” between women at the target age who had and had not performed breast awareness (80.4 versus 77.5, *p*-value = 0.002) and mammogram (77.1 versus 70.3, *p*-value = 0.009) regularly as recommended. Moreover, attitudes towards general health check-ups subscale scores were significantly higher in women who had performed clinical breast examination as frequently as recommended than those who had not.

**Conclusions:**

The research reveals that practical barriers and attitudes towards general health check-ups are important factors to take into account in determining African migrant women’s participation in breast cancer screening. Progress in reducing breast cancer deaths through early detection needs to focus on attitudinal change among African migrants.

## Background

Breast cancer is the leading source of mortality and morbidity among women globally. Its incidence is rising among African women and their mortality rate is significantly higher compared to women in developed countries [1, 2]. Despite the fact that breast cancers in African women present at a younger age and at later stage [3, 4], research has demonstrated that both in their home countries and also in many Western countries, the participation of African women in breast cancer screening measures is poor [5–7].

Due to civil war and unrest in many African countries and also growing economic mobility, an increasing number of African people are migrating to the more developed countries of the world. Australia is a prime example of this trend. Even though there is no recent statistics for the number of Africans in Australia, the Australian Bureau of Statistics (ABS) [8], records shows that in 2006, a total of 248, 699 people born in Africa were living in Australia, compared to 147,876 in 1996, constituting almost 100% increase. Since 2006, more than 50,000 more migrants from Africa have arrived in Australia [9].

However, little is known about the extent to which not only these recent migrants but also those who have been living for a longer period in Australia have made use of cancer screening services. One such service, BreastScreen, is a national population screening program that has been operating in Australia since 1991. It is available, without cost, to women in the 50–74 age bracket, who are encouraged to have a mammogram every two years [10]. While no national data on the mammographic screening rate of African immigrant women exists, studies have shown that women from culturally and linguistically diverse (CALD) backgrounds present themselves for BreastScreen examinations at much lower rates than Australian-born women [11, 12]. This is in line with the low participation rates in breast cancer screening measures reported in numerous international studies among African immigrant women [5, 6, 13]. For example, poor participation pattern was observed in a recent limited study among African-Australian women [5]. There is pressing need for more empirical studies utilising large population samples to gain an in-depth of understanding of cancer screening practices among African women living in Australia. This will help to facilitate the provision of prevention measures that will meet their needs.

An understanding of demographic factors is important in developing effective interventions. One such factor is length of stay in the host country, which previous studies both in Australia and abroad have consistently shown to be significant in determining breast cancer screening behaviours [14–16]. Other important factors have been shown in Australia to include marital status [17] while in the United States ethnicity plays a major role [15]. In Nigeria, Oluwatosin [7] associated BSE and CBE with marital and educational status (7). The demographic indicators for African migrants’ participation in breast cancer screening are yet to be explored. Gaining insight into these demographic factors will enable targeted and effective interventions for promoting breast cancer screening among African migrants.

Available literature on African women’s attitudes to general health check-ups suggest that they do not prioritise their own health [18]. While this suggestion is not conclusive and explanation for the women’s attitude is not easily accessible in the literature, we can draw some observations from a South African study which reported that poverty and low income levels, make black women feel guilty when they spend their money on their own health [19]. It is important to note that Africans are not homogenous and in fact this population is characterised by wide linguistic, cultural and religious diversity. African continent has more than 54 countries with each of the countries having its own diversity. However, there are certain values that are shared. Informed by African culture with its emphasis on communality and connectedness, African women may prefer to put the needs of extended family members before their own. And when it comes to breast cancer, many may dismiss it as a disease of the white population in order to justify their underutilisation of preventive services [20]. Moreover, they may find it difficult to accept and practice the screening concept when they are asymptomatic. The extent to which this attitude is brought to Australia with the African migrant women is yet to be explored.

Evidence shows that decisions on whether to participate in breast cancer screening measures or not is influenced by women’s beliefs about causes, treatment and the outcomes of breast cancer [21, 22]. The poor participation of African women in screening measures has been consistently linked with the influence of cultural factors and also fatalistic beliefs about the cause of breast cancer [6, 23]. In the United Kingdom for instance, breast cancer is considered a taboo topic and stigmatised among African migrants [16]. In the USA, African migrant women regard cancer as a curse inflicted by an evil spirit and they hold an underlying belief that a person diagnosed with cancer must have done something bad to deserve it [13]. Other barriers impeding African migrant women’s participation in breast cancer screening are fear (in relation to pain during screening and also of receiving a cancer diagnosis), stigma and feelings of shame about having to expose their naked body [13, 14]. Research among African migrant women in the USA [13] and the UK [16] reported lack of awareness, low perceived risk of having breast cancer, health care costs, lack of transportation to services providing mammography and also to women having other priorities such as work, school and familial commitments. The belief that their religion will protect them means that African women living in the USA often underutilise screening services [12]. Patriarchal and gender role expectation is another barrier. Low participation of African migrant women in breast cancer screening is reported to be due to either their spouse not consenting to the procedure [13] or that they needed to ask for permission from their husbands before seeking health care [19]. Whether such beliefs are held by African women in Australia is not conclusive but it can be asserted with a fair degree of confidence that promoting breast cancer screening among the growing African population in Australia calls for an understanding of their related cultural beliefs. The purpose of this study is to report breast cancer screening status of African migrant women and factors associated with their breast cancer screening behaviour in Australia.

Identifying factors that predict the practice of breast examination among African migrant women in Australia is important. This will be of help in motivating the women to practice breast cancer screening thereby enhancing early detection and reducing mortality from breast cancer.

## Methods

### Aims

To report breast cancer screening status among African migrant women in Australia; to investigate the association between demographic factors and their breast cancer screening practices and also to investigate the relationship between these women’s cultural beliefs and their breast cancer screening practices.

### Study design and setting

A descriptive, cross-sectional study using a self-administered questionnaire was conducted between October 2013 and December 2014 in Sydney, Australia.

### Recruitment of study participants

A Google search of African community groups, religious organisations and associations in Sydney, Australia was carried out. The groups identified were then approached by the first author, who is of African origin, and asked for permission to recruit potential participants from among their memberships. Personal networking, attendance at African community events and end-of-year activities were also utilised. A convenience sampling technique was used to recruit 264 participants who met the following selection criteria: (1) being a migrant of African descent; (2) being aged 18 years and over (3) being resident in Sydney, Australia and (4) having no history of breast cancer.

### Data collection

Ethics approval was obtained from Institutional human ethics committee. Prior to data collection, the first author as well as some leaders of the organisations and associations, distributed information sheets containing details of the study to members of various organisations and associations who were asked to participate in the study. The researcher explained the study, method of administration, associated risks and benefits of participation and a number of confidentiality issues. The potential participants were given opportunity to ask questions and clarify their thoughts. They were made aware that participation was voluntary and that they would not be identified in research reports. The women were assured that their participation or otherwise do not affect their membership of their organisation or association. The women who agreed to participate in the study were then asked to complete the study questionnaire, a task which took approximately 10–15 min. Any of the consenting participants who (due to their level of English proficiency) requested assistance with explanation of unfamiliar terms or concepts, were provided with additional explanation. Considering that the questionnaire was a mass-distribution one, its return was taken as an indication of their willingness to participate and voluntary consent. The completed surveys were collected from the organisations and associations by the first author.

### The instrument

The African version of the Breast Cancer Screening Beliefs Questionnaire (BCSBQ) was used for data collection [24]. BCSBQ was originally developed in English as a culturally sensitive instrument to assess women’s knowledge about and attitudes towards breast cancer and screening practices. The instrument has subsequently been translated and validated in studies among Chinese [25], Korean [26], Indian [27] and Arabic population groups [28] living in Sydney. However, the wide linguistic diversity of African languages suggested that the African version of the BCSBQ had to be administered in English language. This did not disadvantage the participants because as depicted in the result below, most participants claimed to have good English language proficiency. As stated earlier, in order to ensure the clarity, understanding ability and readability of the instrument, extensive explanation of the core content of the questionnaire was provided. In addition, the instrument was piloted among 15 African Australian women with various demographic backgrounds who unanimously confirmed its comprehensibility. The African version revealed good reliability with Cronbach’s alpha for the three subscales ranging from 0.80 to 0.88 and the validation study has been published elsewhere.

Originally the BCSBQ consisted of three main subscales: 1) Attitudes towards general health check-ups. There were four items under this heading, designed to assess whether, in the absence of signs or symptoms of illness, the respondent routinely presented herself for general health check-ups. 2) Knowledge and perceptions of breast cancer. The four items under this heading were designed to elicit information on cultural beliefs such as fatalism about breast cancer. 3) Barriers to mammographic screening with items such as transportation issues and the need to take off their clothes for mammogram. The five items under this heading were designed to ascertain what the respondent perceived as preventing her from attending mammographic screening.

When the validation study of the African version of the BCSBQ was applied, it was found that it could be conceptualised as a four rather than a three factor model. This was because it was realised that the third subscale, viz, *Barriers* to mammography, was best split into two factors, namely *psychological issues* (such as feelings of fear or embarrassment) and *practical issues,* including lack of English-language proficiency and transport difficulties.

Each of the items within the three main subscales had a 5-point Likert scale ranging from ‘Strongly agree’ (score of 1) to ‘Strongly disagree’ (score of 5) and included a ‘Don’t know’ option. The mean response to the items within a subscale was calculated and then converted to a range from 0 to 100. If a woman scored all items within a subscale as 5, the final score was 100, whereas if a woman scored all items as 1 the final score was 0. A higher subscale score reflects a more positive attitude (Attitude subscale), more knowledgeable (Knowledge subscale) and less perceived barriers to having mammogram (Barrier subscale). Missing values would be imputed by the half-rule, i.e. if more than half of the items in a subscale were validly answered, any missing response in that subscale was imputed by the mean of the answered items, otherwise the subscale was discarded. This rule for computing subscale scores in patient-reported outcome instruments when there are missing values has been widely used and recommended [29, 30]. Floor and ceiling effects were also examined to determine whether the 5-point Likert scale was sufficient to distinguish the responses at the two extremes clearly.

Demographic information including age, length of stay in Australia, English-language proficiency and education level was also collected. The women were further asked if they had ever heard of BSE, CBE and mammography and if so, how frequently they practised any or all of these.

### Statistical analysis

Participants’ demographic and baseline characteristics as well as their breast cancer screening practices were summarized by descriptive statistics. Numbers and proportions of women who had heard of BSE, CBE and mammography and had either performed them at irregular intervals or at the recommended frequency, (BSE monthly, CBE annually and mammograms biennially) were computed. Multivariable logistic regression analyses were performed to investigate the impact of the demographic variables on the likelihood of women in the target age range having the aforementioned screening practices as recommended. An adjusted odds ratio (OR) was reported together with the 95% confidence interval (CI). The three subscale scores of the African version of Breast Cancer Screening Beliefs Questionnaire (BCSBQ) were computed, and the mean scores between participants who did and those who did not engage in the screening practices were compared by t-tests. In an earlier study investigating the psychometric properties of the BCSBQ, it was found that the “Barriers to mammographic screening” subscale needed to be divided into two “daughter subscales” described above and, namely psychological and practical barriers [31] and these were considered and delineated along with the other subscales.

## Results

Demographic and baseline characteristics of the 264 women recruited are summarised in Table 1. Their ages ranged from 18 to 69, with a mean (standard deviation) of 49.5 (10.4) years. They had lived in Australia for a mean of 8.7 (4.9) years. Most were married (66.2%), had attained secondary or tertiary or education qualifications (58.3%), spoke English at home (74.6%) and rated their English level as very good (56.8%).

**Table 1:** Demographic characteristics of participants (*N* = 264)

Characteristic	*N*	(%)
Age (year) (Mean: 49.5, SD: 10.4, missing: *N* = 7)		
19 or younger	1	(0.4)
20–29	10	(3.9)
30–39	32	(12.5)
40–49	77	(30.0)
50–59	92	(35.8)
60–69	45	(17.5)
Country		
East Africa	17	(6.4)
West Africa	95	(36.0)
North Africa	88	(33.3)
South Africa	64	(24.2)
Language at home		
African	18	(6.8)
Dinka	40	(15.2)
English	197	(74.6)
Others	9	(3.4)
Length of stay in Australia (year)		
(Mean: 8.7, SD: 4.9, missing: *N* = 12)		
0–5	70	(27.8)
6–10	111	(44.0)
11–15	52	(20.6)
16–20	12	(4.8)
21–25	6	(2.4)
26 or above	1	(0.4)
Marital status (missing: *N* = 1)		
Single	19	(7.2)
Married/defacto	174	(66.2)
Divorced/separated	60	(22.8)
Widowed	10	(3.8)
Education level		
Primary school	20	(7.6)
Secondary school	47	(17.8)
TAFE/college	43	(16.3)
Tertiary or above	154	(58.3)
Current employment status (missing: *N* = 1)		
Employed, full time	125	(47.5)
Employed, part time	94	(35.7)
Unemployed	38	(14.4)
Retired	6	(2.3)
Self-rated English level		
Little	14	(5.3)
Average	21	(8.0)
Good	79	(29.9)
Very good	150	(56.8)

Most participants (76.1%) had heard of breast awareness and mammography (85.2%). While approximately three-quarters had heard of these measures or had performed them, only 11.4% examined their own breasts monthly. Meanwhile, 65.9% within the target age group i.e. between 50 and 74 years had had a mammogram at the recommended bi-annual frequency. However, for clinical breast examination, less than half of the participants heard of clinical breast examination. Although 73 (64.6%) of the 113 women who heard of clinical breast examination had ever practiced it, only 4 (3.5%) had performed it as recommended. The proportion within the target age group (40 years or older) was practically no difference (3.3%) (Table 2).

**Table 2:** Breast cancer screening practices

Screening practice	All participants		Target age group^a^	
	*N*	(%)	*N*	(%)
Breast awareness				
Ever heard of it	201/264	(76.1)		
Ever preformed	154/201	(76.6)		
Performed as recommended (monthly)	23/201	(11.4)		
Clinical breast examination				
Ever heard of it	113/264	(42.8)	92/214	(43.0)
Ever preformed	73/113	(64.6)	63/92	(68.5)
Performed as recommended (annually)	4/113	(3.5)	3/92	(3.3)
Mammogram				
Ever heard of it	225/264	(85.2)	126/137	(92.0)
Ever preformed	168/225	(74.7)	102/126	(81.0)
Performed as recommended (biennially)	123/225	(54.7)	83/126	(65.9)

^a^Clinical breast examination: 40 years or older; Mammogram: between 50 and 74 years

Table 3 shows the impact of the demographic characteristics on the likelihood of regularly performing breast awareness and mammogram as recommended within the target age group revealed by multivariable logistic regression analyses. Since only 3 participants in the target age group practiced clinical breast examination as recommended, it was expected that the estimates would associate with a large standard error and thus no significant results would be obtained. Therefore, the corresponding analysis was omitted. For monthly breast awareness, based on all participants who ever heard of it, women who came from South Africa (OR = 3.25, 95% CI = 1.01 to 10.41, *p*-value = 0.031) were significantly more likely to pay attention to their breast than those from West Africa, while those who came from North Africa were least likely (OR = 0.67, 95% CI = 0.14 to 3.19, *p*-value = 0.151). For mammogram, based on those aged between 50 and 74, age and employment status were found to be influential factors: older women (OR = 1.20, 95% CI = 1.06 to 1.35, *p*-value = 0.003) were more likely to have a mammogram every two years than younger women, while unemployed or retired women (OR = 0.25, 95% CI = 0.07 to 0.89, *p*-value = 0.033) were less likely than the employed counterpart.

**Table 3:** Demographic characteristics and breast cancer screening practices among those who have heard of the practice and within the target age group

Variable	Breast awareness as recommended (monthly) (*N* = 201)			Have mammogram as recommended (biennially) (Target age group^a^ *N* = 126)		
	OR	95% CI	*P*-value	OR	95% CI	*P*-value
Age (year)	0.99	(0.95, 1.04)	0.829	1.20	(1.06, 1.35)	0.003
Length of stay in Australia (year)	1.04	(0.95, 1.14)	0.380	1.03	(0.93, 1.14)	0.562
Country						
West Africa	1			1		
North Africa	0.67	(0.14, 3.19)	0.151	1.15	(0.38, 3.49)	0.551
East Africa	1.81	(0.29, 11.37)	0.011	0.80	(0.13, 4.94)	0.836
South Africa	3.25	(1.01, 10.41)	0.031	0.76	(0.24, 2.39)	0.653
Marital status						
Married/defacto	1			1		
Single/divorced/separated/widowed	1.00	(0.35, 2.80)	0.993	0.99	(0.39, 2.48)	0.978
Education level						
Tertiary or TAFE/college	1			1		
Primary or secondary school	1.03	(0.28, 3.74)	0.968	0.60	(0.22, 1.63)	0.320
Current employment status						
Employed, full time or part time	1			1		
Unemployed/retired	1.49	(0.33, 6.70)	0.602	0.25	(0.07, 0.89)	0.033

*Abbreviations*: *OR* adjusted odds ratio, *CI* confidence interval

^a^Between 50 and 74 years

The mean and standard deviation of the subscale scores of the African version of BCSBQ classified by breast cancer screening practices are shown in Table 4. All participants answered all items in the Attitude subscale. Missing values were imputed based on the half-rule for one participant who answered only three items in the Knowledge subscale and another three participants who answered only three or four items in the Barrier subscale. However, two participants who answered fewer than two items in the Barrier subscale were excluded from the analysis. Significant different scores were found in the “Practical barriers” between women at the target age who had and had not performed breast awareness (80.4 versus 77.5, *p*-value = 0.002) and mammogram (77.1 versus 70.3, *p*-value = 0.009) regularly as recommended. Moreover, the “Attitudes towards general health check-ups” subscale scores were significantly higher in women who had performed clinical breast examination as frequently as recommended than those who had not (81.3 versus 43.8, *p*-value = 0.016).

**Table 4:** Mean (standard deviation) of subscale scores of the African version of Breast Cancer Screening Beliefs Questionnaire by breast cancer screening practices

	Ever preformed			Performed regularly as recommended^a^		
	Yes	No	*P*-value	Yes	No	*P*-value
*Breast awareness*						
Attitudes towards general health check-ups	45.5 (26.4)	37.6 (27.8)	0.079	42.7 (33.6)	43.8 (26.0)	0.851
Knowledge and perceptions about breast cancer	49.3 (27.4)	40.4 (26.3)	0.051	44.3 (30.3)	47.6 (27.0)	0.587
Barriers to mammographic screening	58.9 (15.8)	55.1 (16.7)	0.167	63.5 (19.0)	57.3 (15.5)	0.082
Psychological barriers	44.8 (23.5)	43.7 (20.3)	0.768	52.2 (26.3)	43.6 (22.1)	0.089
Practical barriers	79.5 (11.4)	72.2 (19.2)	0.089	80.4 (15.5)	77.5 (13.7)	0.002
*Clinical breast examination* (*40 years or older*)						
Attitudes towards general health check-ups	48.6 (25.2)	37.1 (28.8)	0.055	81.3 (18.8)	43.8 (26.2)	0.016
Knowledge and perceptions about breast cancer	50.6 (24.4)	36.4 (24.0)	0.011	61.1 (12.7)	45.6 (25.3)	0.296
Barriers to mammographic screening	58.2 (16.2)	54.3 (18.3)	0.314	70.0 (22.9)	56.5 (16.7)	0.176
Psychological barriers	45.1 (23.3)	42.0 (20.6)	0.541	63.9 (33.7)	43.5 (21.9)	0.122
Practical barriers	77.8 (13.6)	72.8 (24.1)	0.209	79.2 (19.1)	76.1 (17.5)	0.770
*Mammogram* (*between 50 and 74 years*)						
Attitudes towards general health check-ups	40.4 (26.0)	31.3 (22.1)	0.113	38.9 (25.5)	38.2 (25.7)	0.884
Knowledge and perceptions about breast cancer	39.2 (25.7)	30.2 (22.2)	0.116	36.7 (24.7)	39.1 (26.4)	0.611
Barriers to mammographic screening	54.2 (13.9)	52.7 (12.0)	0.637	55.1 (12.5)	51.6 (15.2)	0.178
Psychological barriers	39.8 (19.0)	40.6 (17.3)	0.844	40.4 (18.1)	39.1 (19.9)	0.730
Practical barriers	75.7 (14.1)	70.8 (13.1)	0.122	77.1 (11.7)	70.3 (16.8)	0.009

^a^Breast awareness, monthly; Clinical breast examination, annually; Mammogram, biennially

## Discussion

Our study explored breast cancer screening practices of African migrant women in Australia and provided insights into the relationship between these practices and demographic factors. This was the first cross-sectional study of its kind in Australia.

Our findings revealed that while the majority of the participants had heard about breast awareness (76.1%) and mammography (85.2%), only 11.4% actually practised monthly breast awareness. This is the first time that we are hearing about this low monthly attention of African migrant women to their breast in Australia. However, this finding is consistent with previous findings of studies among other migrant populations reported in international literature [32]. It also echoed the findings of an earlier study of Indian-Australians [17]. This may be explained by the high knowledge deficit about breast awareness common among African women and their negative attitudes towards discussions about and the touching of reproductive organs [15]. The low participation of African migrant women in breast awareness is of huge concern when one considers what it would have cost Australian government in initiating the BreastScreen. Targeted strategies for raising breast cancer screening awareness and participation among African migrant community in Australia need to be identified. This can be in terms of oral presentations on the need for women to be aware of their breast should be made during events attended by these migrants. Interventions for attitudinal changes towards breast cancer screening among this cohort of Africans in Australia and other developed countries hosting African migrants is suggested.

A much smaller proportion of this cohort had heard of CBE (42.8%) and only 3.3% performed it as recommended. Even though low participation rates have been reported among other migrant populations in Australia [17] and the USA [14], this appears to be one of the lowest rates among CALD groups in Australia [11, 12]. One possible explanation could be the participants’ recent arrival in Australia, particularly those from the Eastern, Western and Northern regions of Africa [33] . Other reasons could be that these women are not familiar with such measures and that perhaps opportunities for CBE are not available in their countries of birth [6].

Nevertheless, that our results showed that nearly two third of the participants (65%) had had mammography is promising. This participation rate is higher than the national figure of 55% among Australian-born women and 50% among women from non-English speaking backgrounds [11, 34]. This may be due to the recruitment setting of this study. In their study of breast cancer screening behaviour in the USA, Millon-Underwood and Kelber [35] suggested that factors such as the participants’ residence in areas with easy access to breast cancer screening facilities can influence screening behaviour. It is significant that the African women in the current study were recruited from densely populated Sydney with its greater access to BreastScreen services compared to those in rural or remote areas. Further study with wider cover of sample recruitment is required to investigate this phenomenon and further validate this promising finding.

Our study revealed significant contrasts in the screening behaviour of those women who had participated in breast cancer screening and those who had not. This finding is consistent with those of studies among African migrant women in the USA [13] and among Indian-Australian women [17]. The failure of the latter to perform breast awareness exercises, particularly mammography, may be attributable to fears of the unknown in terms of finding cancerous lumps. This suggests the need for the generation and provision of culturally sensitive information and interventions to change this kind of thinking. Strategies aimed at alleviating the fear of the unknown should be taught among migrant population. Considering that religious gatherings were among avenues used in recruiting participants for this study, we suggest ongoing strong collaboration and partnership with religious organisations. Possible slogans such as the “devil you know is better than the devil you do not know” can be used in motivating and increasing breast cancer screening among African migrants.

In terms of demographic characteristics, our findings showed that the region of African continent where the participants originated from and also their age and employment status, were significant factors associated with their breast cancer screening practices. Our findings imply that a program to raise breast cancer screening among African women cannot assume that they are a homogeneous group. For instance, our study showed that migrant women from South Africa are more likely to perform breast awareness than women from West Africa and North Africa. This reason may be that particularly in its urban areas, South Africa has a more highly developed health system than other African countries and provides more plentiful breast screening services.

Another factor is that women from South Africa tend to have a longer migration history than those from other parts of Africa. This is in line with a body of literature suggesting that the longer a migrant stays in a country, the more chance that they will be exposed to available health information such as breast cancer screening and as a result adopt new health behaviours [13, 32]. Most recent North African migrants living in Australia arrived as refugees and may be preoccupied with issues that relate to their forceful uprooting from their home countries. Settling into a new country with a totally different culture, may account for the very low rate of breast awareness practice among them. While our study identified North African migrant women as being in most urgent need of breast awareness interventions, there is also a need to raise the levels of breast cancer screening awareness among African migrants in Australia generally. This in fact also applies to those western countries which have significant African migrant populations.

The relationship observed between employment status and screening behaviours was inconsistent with the findings of studies among women from Arabic and Indian background [11, 17] living in Australia. Our study findings indicated that unemployed or retired women were significantly less likely to participate in mammography when compared to employed women. This could be due to working women having more exposure to health information and therefore possessing greater knowledge about access to local screening services. It is also possible that the unemployed are relatively newer arrivals and are preoccupied with other settlement issues. Unlike unemployed and retired Arabic women in Australia who were reported to have utilised the available time at their disposal to increase their mammographic practice [11], our study did not find the time available to unemployed and retired African women was of any particular advantage in terms of breast cancer screening behaviour.

However, one significant finding was that older African migrant women in Australia were more likely to participate in mammography than their younger counterparts. To the best of our knowledge, this finding of a clear association between age and participation in breast cancer screening among African women is the first of its kind in Australia. While previous studies on breast cancer screening among African women reported the age of study participants [6, 7], their findings have not suggested any link between age and breast cancer screening. The findings of our study were not surprising given that advancing age brings with it a higher risk of breast cancer. However, a growing literature indicates that breast cancer is more common among young African women [1, 2] than among women of similar age in other ethnic groups. This in turn indicates that any effort to increase the participation rate of breast cancer screening among African migrants in western countries, demands a greater focus on younger women. Thus health promotion campaigns and services need to be age-appropriate and include images of young people to address the misconception that younger women are immune to breast cancer.

The findings from the subscales examining the relationship between cultural beliefs and migrant African women’s screening behaviours indicate that the concept of having regular health check-ups has a significant impact on those who did or did not have CBEs as recommended. This is because our study indicates that among African migrants the concept of participating in cancer screening measures when according to a woman’s own assessment, she is asymptomatic and otherwise healthy, is foreign or irrelevant. This finding is in line with that of studies among women from other cultures [16, 36, 37]. For breast cancer screening promotion to be effective in the African migrant community, it is important to emphasise the message that “early detection saves lives” in a culturally appropriate manner.

Interestingly, our study demonstrated that practical barriers such as transportation and language rather than psychological barrier such as embarrassment are influential factors that impact on their mammographic screening behaviours than they do among other CALD women living in Australia. Even though African migrant women in this study were equipped with good English proficiency, (more than two thirds of them indicated having either good or very good English proficiency), several factors hindered their participation in breast cancer screening. One is that they may find it difficult to communicate during medical consultations. Another factor, consistent with other studies, could be that transportation difficulties prevent women from travelling to mammographic screening sites [32]. In addition to organising the transportation, the cost of transportation to the venue of the screening can be an issue. We suggest that health authorities explore the possibility of stationing mobile BreastScreen facilities in or near to places that migrants frequently access such as migrant resource centres and adult education centres.

### Limitations

Firstly, an identified limitation of this study is that participants were drawn from a convenience sample of African women who patronise migrant resource centres or those who attend community events. This means that those who are isolated, kept to themselves or have become established enough to stop accessing the migrant resource centres, are likely to be underrepresented in the study. Despite this limitation, the study indicates a need for more inclusive strategies that reach larger African migrant women population.

Secondly, the study utilized self-reported measures of breast cancer screening practices that may not be accurate. Further studies designed to ensure adequate verification of self-reported information are warranted.

## Conclusions

Given the Australian government’s extensive breast cancer screening programs, the participation of African Australian women in this study can be enhanced. This study contributes to the growing literature on breast cancer screening practices of migrant and culturally diverse population. The findings indicate that a number of barriers to breast cancer screening such as fatalism and fear of the unknown which may not be considered in developed countries have significant impact on breast cancer screening behaviour of migrant population. The general health seeking behaviour and attitudes towards general health checks are important issues determining compliance and adherence with recommended breast cancer screening practices. There is need for nurses, community health providers and other health providers working with African migrant women in Australia to initiate targeted interventions aimed at improving the women’s participation in breast cancer screening. Religious organisations and gatherings were sources of recruitment for this study, collaboration and ongoing partnership with religious leaders may progress preventative strategies aimed at reducing breast cancer deaths. Information materials and information targeted at various women age groups will be beneficial in promoting breast cancer screening among young, mid-life and older women.

## Abbreviation

BCSBQ: Breast cancer screening beliefs questionnaire
